# On the Relation Between the Interstimulus Intervals and Multi-Muscle nTMS Motor Mapping

**DOI:** 10.1007/s10548-025-01128-9

**Published:** 2025-07-30

**Authors:** Anastasiia Asmolova, Anastasiia Sukmanova, Milana Makarova, Pavel Novikov, Vadim Nikulin, Maria Nazarova

**Affiliations:** 1 Max Planck School of Cognition, Leipzig, Germany; 2 Department of Neurology, Max Planck Institute for Human Cognitive and Brain Sciences, Leipzig, Germany; 3 HSE University, Centre for Cognition and Decision Making, Moscow, Russia; 4 HSE University, Center for Bioelectric Interfaces, Moscow, Russia

**Keywords:** Motor cortex, nTMS motor mapping, Motor evoked potentials, Interstimulus interval

## Abstract

Although the interstimulus interval (ISI) is one of the crucial parameters in the transcranial magnetic stimulation (TMS), the ISI effect on the results of the TMS motor mapping is usually overlooked. This study explored the influence of ISI, ranging from 1.5 to 41 s, on multi-muscle navigated TMS (nTMS) motor mapping results. Twenty-six healthy male volunteers underwent four nTMS motor mapping sessions on two separate days. We mapped the muscles' cortical representations (MCRs) of the five upper limb muscles: abductor pollicis brevis (APB), abductor digiti minimi (ADM), first dorsal interosseous (FDI), extensor digitorum communis (EDC), and biceps brachii (BB). We estimated the relationship between ISIs and trial-to-trial motor evoked potentials (MEPs) amplitudes and MCR areas. In addition, we accounted for the association between the ISI and TMS mapping procedure parameters such as the distance between the successive stimulation points, the number of stimuli in a TMS session, and the stimulus counting number. A weak positive association was observed between: (1) trial-to-trial ISI and MEP amplitude and (2) median ISI and MCR areas. We recommend reporting ISI values in TMS motor mapping studies and monitoring the impact of ISI on MEP amplitudes.

**Supplementary Information:**

The online version contains supplementary material available at 10.1007/s10548-025-01128-9.

## Introduction

Navigated transcranial magnetic stimulation (nTMS) is widely used to non-invasively modulate the brain in healthy and clinical populations for research, diagnostic, and treatment purposes. The interval between the successive stimuli during a TMS session – the so-called interstimulus interval (ISI) – is one of the most critical parameters for repetitive TMS (rTMS) and paired-pulse TMS protocols, where different ISIs are supposed to result in the opposite neuromodulatory effects (Vucic et al. 2006). ISI duration has also been reported to influence the stimulation effects during single-pulse TMS protocols applied to a single cortical spot (Julkunen et al. 2012; Vaseghi et al. 2015; Hassanzahraee et al. 2019; Kallioniemi et al. 2022). Thus, in general, longer ISIs were associated with bigger motor-evoked potential (MEP) amplitudes (Julkunen et al. 2012; Vaseghi et al. 2015; Hassanzahraee et al. 2019; Matilainen et al. 2022). Also, single-pulse TMS output while using longer ISIs (up to 15 s) was reported to be more reliable compared to TMS output while using shorter ISIs (Hassanzahraee et al. 2019; Kallioniemi et al. 2022).

Apart from stimulating one cortical spot, TMS is widely used for cortical mapping (Romero et al. 2011; Krieg et al. 2017; Pitkänen et al. 2018) when the maximal stimulation location is moved over the skull. In TMS motor mapping, stimuli are delivered every 2–20 mm with simultaneous electromyography (EMG) recording from the target muscles (Krieg et al. 2017; Raffin and Siebner 2019; Nazarova et al. 2021). The recorded MEPs are then used to create muscle cortical representations (MCRs) (De Carvalho et al. 1999; Novikov et al. 2018). Even though nTMS motor mapping has been approved by Food and Drug Administration for presurgical brain mapping for more than a decade (Krieg 2017) and it is widely used to assess cortical reorganization (Traversa et al. 1997; Liepert et al. 2000; Van De Ruit and Grey 2017; Bulubas et al. 2018; Raffin and Siebner 2019), its methodology is still highly variable. Among different issues, the role of the ISI during mapping remains unclear (Sondergaard et al. 2021). Thus, in the recent study on TMS motor mapping methodology, the authors emphasized using ISIs not shorter than 5 s “to avoid facilitation or depression of the MEP amplitude” (Weise et al. 2023). In addition, many TMS studies use 5 s as a “safe” ISI not to induce carry-over effects (Fecchio et al. 2017; Hannah et al. 2018; Hernandez-Pavon et al. 2023).

At the same time, studies specifically investigating the influence of ISI during TMS motor mapping primarily compared ISIs shorter than 5 s, namely 1.5 and 4-s ISIs (Van De Ruit et al. 2015; Cavaleri et al. 2018). Thus, in one of the first papers on the topic, ISIs that are much shorter than 5 s – 1.5 s – were recommended as optimal (Van De Ruit et al. 2015). Notably, the main finding of this paper was a lack of difference in the centers of gravity (CoG: the size and the amplitude-weighted centre of the muscle cortical representation) locations between the maps obtained during TMS mapping sessions with shorter and longer ISIs. However, it has been previously shown that CoG for TMS motor mapping is not the most sensitive parameter (Nazarova et al. 2021), so the fact that CoG was not dependent on ISI does not exclude the ISI influence on other, more sensitive MCR parameters. At the same time, in a later work, good to excellent relative reliability was also shown for MCR areas and volumes for TMS mapping performed using either 2 or 4-s ISIs (Cavaleri et al. 2018). Yet in another work, absolute reliability for MCR areas and volumes was similar for the MCRs obtained using 2-s ISIs compared to MCRs obtained using 7-s ISIs (Jonker et al. 2019). And again, a short ISI (2 s) was recommended. In clinical nTMS motor mapping, a protocol with ISI of 4–5 s from Raffin et al. 2015 was recommended (Krieg et al. 2017). However, it should be noted that many clinical TMS motor mapping studies do not report ISI at all (Traversa et al. 1997; Krause et al. 2006; Krieg et al. 2015; Lam et al. 2019) and do not discuss it among the important parameters (Sollmann et al. 2021; Umana et al. 2021). Novel technological achievements such as robotically assisted TMS (Kantelhardt et al. 2010; Harquel et al. 2016; Giuffre et al. 2021; Kahl et al. 2023) and multi-locus TMS (Koponen et al. 2018; Nieminen et al. 2022; Navarro de Lara et al. 2023) provide a way of routine use of such short ISIs. To our knowledge, all recent works dedicated to robotically-assisted TMS mapping used 5-s ISIs or shorter (Kantelhardt et al. 2010; Harquel et al. 2016; Grab et al. 2018; Giuffre et al. 2021; Kahl et al. 2023), in some works, the ISIs were shortened to 1 s (Grab et al. 2018). Thus, the question of whether ISI duration is important for TMS motor mapping remains open.

Currently, the importance of ISI is often overlooked in single-pulse TMS studies, including TMS motor mapping research. For instance, the checklist of factors to be reported in single-pulse TMS studies omits ISIs (Chipchase et al. 2012). To systematically assess the effect of ISIs on the motor mapping results, in this study, we aimed to probe the relationship between ISIs ranging from 1.5 to 41 s and one of the most commonly used TMS motor mapping outputs — MCR areas — during a comprehensive manual nTMS motor mapping of multiple upper limb muscles similar to the one we used to study MCR parameters reliability (Nazarova et al. 2021). We hypothesized that an increase in ISI up to 41 s may still lead to an increase in MEP amplitudes and MCR area sizes. For this, we estimated the relationship between trial-by-trial MEP amplitudes and ISIs, the link between MCR areas and median ISI. In addition, in our analysis, we accounted for the TMS mapping procedure confounds, such as the number of stimuli and the distance between the stimulation points.

## Materials and Methods

### Participants

Twenty-six young, healthy male volunteers (age 18–36 years old, mean = 23.68, SD = 4.88) participated in the study. The majority of the subjects had not had TMS before; ten subjects were totally naive about TMS. We excluded subjects with a history of neurological/psychiatric disorders, cardiac implants, metallic implants in the head, any regular medication intake, implanted pumps, stimulators, and shunts. We also excluded subjects with special motor skills (such as musicians or surgeons). All volunteers were right-handed, according to their self-report. This study received approval from the HSE University Committee for Interuniversity Surveys and Ethical Assessment of Empirical Research (Protocol No. 75, dated 15.11.2021), and all subjects gave their written consent after being informed of the nature and purpose of the study.

### Overall Procedure

**Fig. 1:**
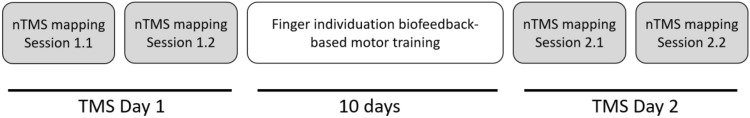
The overall experimental procedure. On Day 1, participants underwent two nTMS mapping sessions (1.1 and 1.2). After ten days of motor training, volunteers underwent the other two nTMS mapping sessions (2.1 and 2.2) on Day 2

The current work is a part of a bigger study investigating motor cortex reorganization during motor learning. Each volunteer underwent two nTMS mapping sessions separated by roughly two weeks (Fig. 1). Within this period, participants underwent ten sessions of finger independence training using visual EMG-based biofeedback.

### Electromyography

**Fig. 2:**
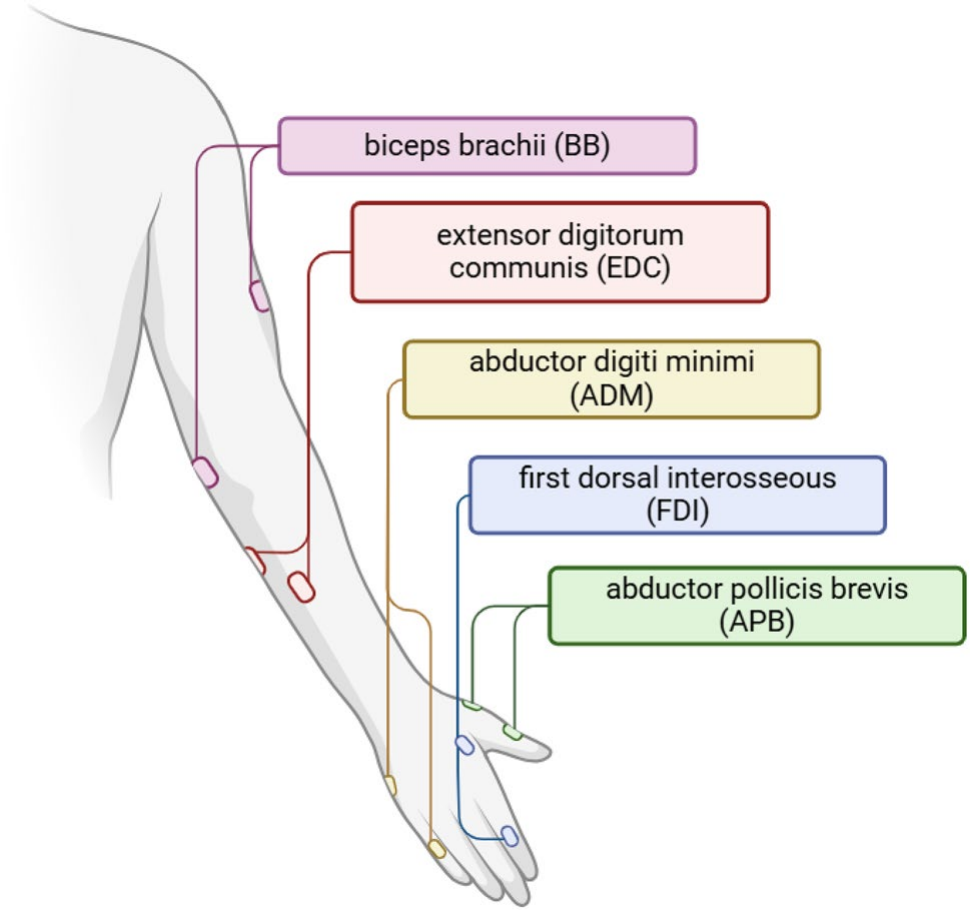
The EMG electrode placement

MEPs were obtained from five right upper limb muscles: abductor pollicis brevis (APB), abductor digiti minimi (ADM), first dorsal interosseous (FDI), extensor digitorum communis (EDC), and biceps brachii (BB) using the integrated EMG device of the eXimia system (3 kHz sampling rate, band-pass filter of 10–500 Hz, Fig. 2). Bipolar surface Ag/AgCl electrodes (0.6-cm2, 3 M Red Dot) were placed using a belly-tendon montage, and the ground electrode was always on the right wrist. The MEP peak-to-peak amplitudes were estimated automatically using the in-built eXimia software (3 kHz sampling rate, band‐pass filter of 10–500 Hz) and rechecked manually by an operator. Noisy trials with pre-stimulation EMG amplitudes higher than 20 μV—the lowest MEP amplitude that could be separated from the noise of an EMG device (Machetanz et al. 2021; Memarian Sorkhabi et al. 2022), peak-to-peak during 1 s before the stimulation, were excluded.

### Structural MRI for nTMS Navigation

On a separate day, before the TMS mapping, individual anatomical T1-weighted magnetic resonance images for their use during nTMS were acquired using a 1.5 T MR-scanner Optima CT 660 scanner, GE Medical Systems (structural T1-weighted images, 1 mm isotropic voxel).

### Multi-Muscle nTMS Mapping Procedure

All TMS procedures followed the TMS safety guidelines (Rossi et al. 2021). Single pulse TMS was performed using a figure-of-eight induction coil (outer winding diameter 70 mm) for biphasic stimulation, connected to an eXimia magnetic stimulator (Nexstim Ltd., Helsinki, Finland, version 5.2.3). eXimia NBS navigation system (Nexstim Ltd.) was used for individual MRI-guided TMS navigation. The nTMS mapping procedure was adapted from Nazarova et al. 2021 and performed by four well-trained operators (AA, AS, MM—trained by MN, and MN herself). As a first step, “rough mapping” was performed (Krieg 2017; Nazarova et al. 2021) to determine the cortical spot, stimulation of which induced MEPs of 500–800 µV from APB. Then, the resting motor threshold (RMT) of APB muscle was determined using the in-built RMT determination algorithm of the eXimia NBS navigation system (Nexstim Ltd.). For the mapping, the stimulation intensity was set to 110% RMT of APB. TMS pulses were delivered using the auto-generated 5 × 5 mm2 square grid from the navigation software using pseudo-random order (moving from the “hotspot” area in the hand knob region to the “outer bounds”). The boundaries of the maps were set if the two consecutive stimuli did not produce an MEP from at least one of the muscles (Nazarova et al. 2021). For every subject, 43–150 stimuli were delivered within the first TMS session during Day 1 (session 1.1) and then repeated one more time during the same day (session 1.2). For each stimulus, the TMS operators had to position the coil to the same location from session 1.1 using the navigation system, proceeding stimulus-by-stimulus. On Day 2 (sessions 2.1 and 2.2), the stimuli from session 1.1 were repeated in the exactly same manner. Although some MCR parameters, like COG, may be reliably obtained using about 60 stimuli (Van De Ruit et al. 2015), there is evidence that the accuracy of other MCR parameters, such as area, volume, and CoG continues to increase with a higher number of stimuli (Nazarova et al. 2021; Sinitsyn et al. 2019).

The experimental sessions 1.1 (1.2) and sessions 2.1 (2.2) for the same subject were conducted at the same time of day ($\pm$ 2 h). At least two experimenters participated in each experimental session. The first researcher performed the stimulation, the second one monitored the subject’s vigilance. The nTMS motor mapping sessions lasted 2–3 h.

### Muscle Cortical Representation Area Calculation

**Fig. 3:**
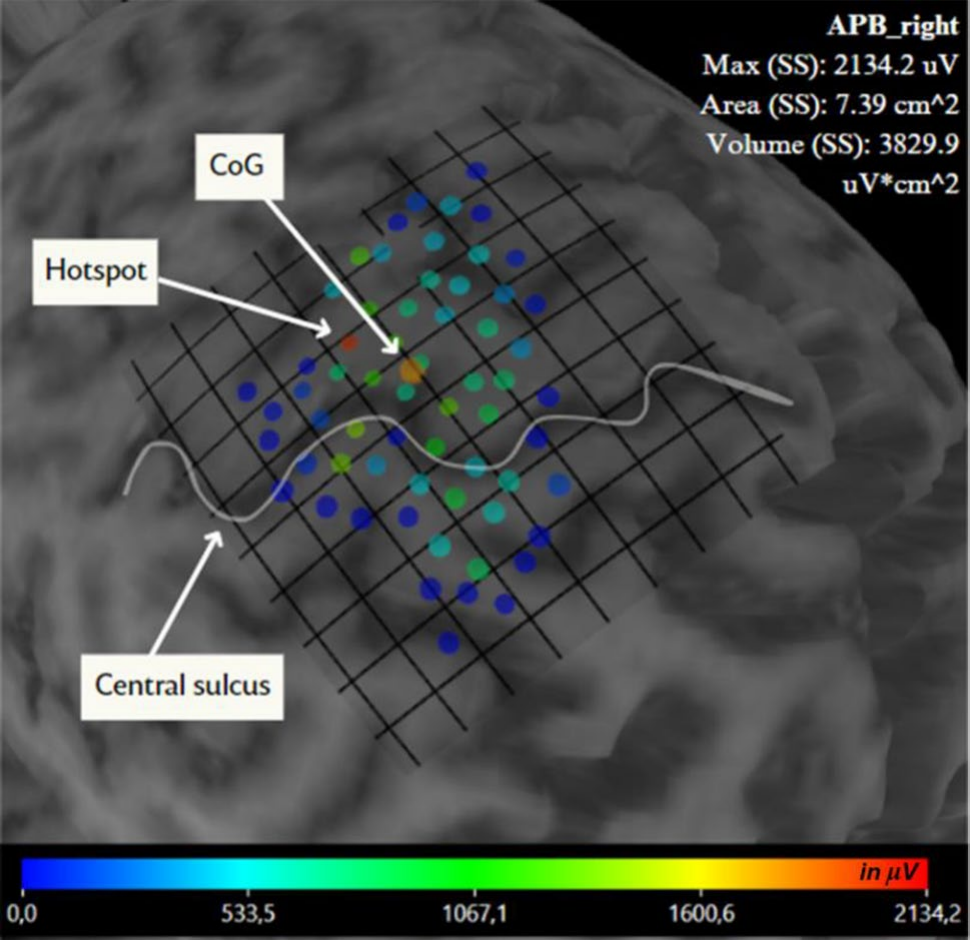
The MCR of the APB in a representative subject is visualized in TMSmap software (Novikov et al. 2018). Colored dots represent the stimulation points. The color reflects the peak-peak APB MEP amplitude in μV (see the color bar), so the red dot represents the hotspot. The big orange dot represents the CoG. The white line marks the central sulcus on the individual MRI. The overlaid grid has 5 × 5 ${mm}^{2}$ square size. The APB MCR area and volume are shown in the upper right corner

MCR areas were constructed using TMSmap software (Novikov et al. 2018). In case there were stimulation points closer than 2 mm apart, they were merged (amplitudes averaged) to one stimulation point in the TMSmap software, and mean MEP amplitudes were used for MCR construction (Fig. 3). The threshold for the mean MEP amplitude (for a merged point) for MCR construction was 50 μV peak-to-peak. MCRs were transformed to a common Montreal Neurological Institute space, using an algorithm described earlier (Nazarova et al. 2021) using SPM8 (The Wellcome Department of Imaging Neuroscience, Institute of Neurology, University College London, UK) and TMSmap (Novikov et al. 2018). For further analysis, we concentrated on the MCR area parameter, for which we have recently reported high relative and absolute test–retest reliability using the same TMS mapping approach (Nazarova et al. 2021).

### Interstimulus Interval Calculation

In manual TMS mapping, the ISI is primarily defined by the time it takes for the operator to move the coil from one stimulation point to another and align it according to the desired location and orientation. Since the navigation system records the times when the stimuli were delivered, ISI for each trial can be calculated as a time difference between the current stimulus time and the preceding one (${time}_{n}- {time}_{n-1}$ where n is the current stimulus number).

If a stimulus was excluded from further analysis during the postprocessing, ISI was calculated for the following stimulus before its exclusion, thus retaining the original ISIs. Each TMS session's first and last stimuli were excluded from the analysis.

### Statistical Analysis

Statistical analysis was performed using R (ver. 3.5.1, RStudio, Inc., Boston, Massachusetts, USA) and MATLAB (ver. R2014b, The MathWorks Inc., Natick, Massachusetts, United States). The distributions of the obtained data (including distributions of ISIs, distances between the two successive TMS stimulation points, MCR areas, and peak-to-peak MEP amplitudes) were tested for normality using the Shapiro–Wilk test. We used non-parametric statistical tests for non-normally distributed data. The distributions of the trial-by-trial ISIs, peak-to-peak MEP amplitudes, and distances between the two successive stimulation points were limited to ± 2 SD and log-transformed for further analysis.

To check the relationship between trial-by-trial ISI and MEP, linear mixed-effect models were used (the trial-by-trial ISI was a fixed effect, the subject was a random effect, and trial-by-trial peak-to-peak MEP amplitude was the outcome variable) for each muscle separately. Different muscle representations were analyzed using separate models, as they are not spatially identical in the cortex, resulting in distinct stimulation target datasets. In addition, we performed the same analysis, excluding the short ISIs from the models, to verify whether the short ISIs (less than 5 s) were driving the effect. To estimate the effect size, Cohen’s ${f}^{2}$ was calculated using marginal (fixed effect) and conditional (fixed and random effects) ${R}^{2}$ from the models (Nakagawa & Schielzeth 2013), where ${f}^{2}$= 0.02 corresponds to a small effect size, ${f}^{2}$= 0.15 corresponds to a medium effect, and ${f}^{2}$= 0.35 to a large one. We also checked the change in significance level of the models, limiting ISI from 40 to 5 s using 5-s steps. As an additional control analysis, we also tested the relationship between trial-by-trial MEP and ISI, removing the muscle-specific effect. We used the combined dataset for all four muscles, excluding trials where any of the four muscles had a zero response. We then z-scored the MEP amplitudes and performed principal component analysis (PCA), selecting the 1 st component explaining the largest variance. The 1 st component was then used together with the trial-by-trial ISI for Spearman’s correlation analysis.

To address the issue of multiple comparisons and examine the simultaneous activation of four muscles, we performed a permutation test with 10,000 repetitions. The analysis incorporated a single-factor mixed regression model, consistent with the model used to investigate the relationship between ISI and MEP in the manuscript. The dataset for this analysis included only TMS stimuli that elicited MEPs in all four investigated muscles. Using the dataset with the MEPs from all the stimuli would not be appropriate for this analysis, as the topography of cortical muscle representations would significantly influence it.

The hotspot for APB was chosen as the starting point for motor mapping in this study, while hotspots of the other muscles (especially ADM and EDC) were located at a distance from it (as it was shown in earlier studies dedicated to intrinsic upper limb muscles TMS mapping, e.g. Raffin et al. 2015; Nazarova et al. 2021). The strongest MEPs for ADM and EDC corresponded to longer ISIs.

To further account for possible confounds that might contribute to the ISI effect on MEP, the linear mixed-effect model was used to assess whether the spatial distance between the successive stimuli influenced the ISI (the distance between two successive stimuli points was a fixed effect, the subject was a random effect, and trial-by-trial ISI was the outcome variable). Then, for non-normally distributed data, Spearman's rank correlation coefficient was calculated for trial-by-trial ISIs and distances between the two successive stimulation points; ISIs and a number of stimuli in a TMS session; trial-by-trial ISIs and peak-to-peak MEP amplitudes.

The relationship between median ISI and MCR area for each mapping session for both days was addressed with a Spearman’s rank correlation coefficient using the permutation test with 10,000 permutations. Additionally, to restrain the effect of confounds on ISI and MCR area relationship, partial Spearman's rank correlation coefficient was calculated for MCR areas and median ISIs to control for the effect of the number of stimuli in each session and the distance between two successive stimulation points.

**Fig. 4:**
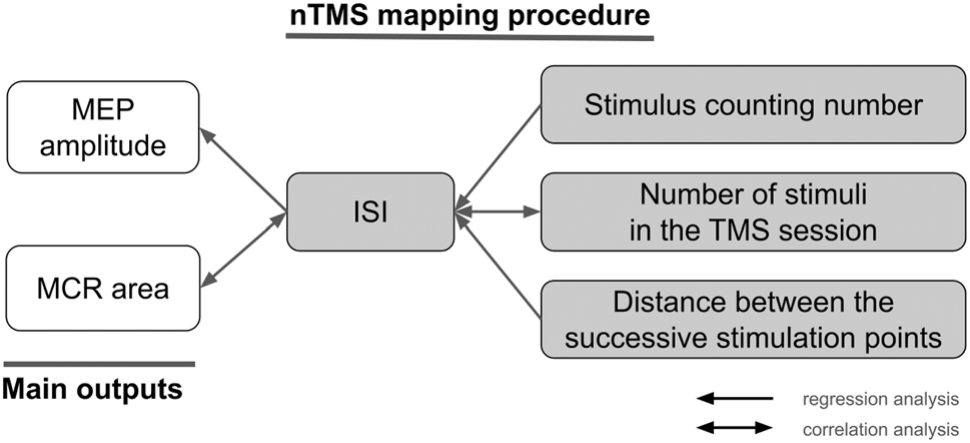
A scheme showing the investigated relationships between the TMS mapping parameters and outputs. ISI stands for interstimulus interval, MEP for motor evoked potential, and MCR for muscle cortical representation. One-sided arrows indicate the direction from the independent to the dependent variable in the regression analysis, and double-sided arrows indicate the correlational relationship between the variables

The general view of the investigated parameters and their relationships is shown in Fig. 4.

## Results

### Descriptive Statistics

#### ISI Range and Normality Check

**Fig. 5:**
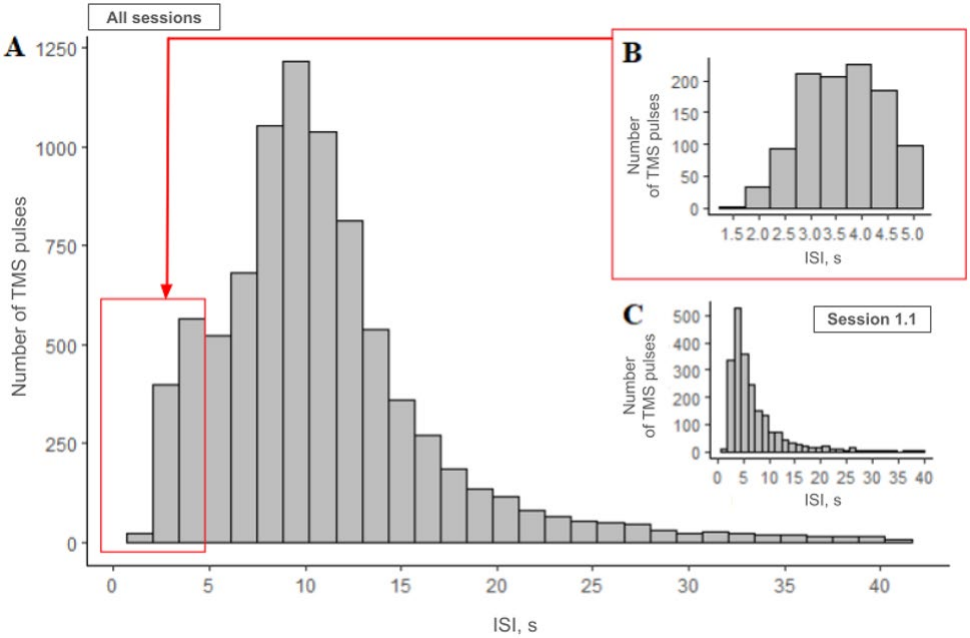
The ISI distribution for all the trials across 26 participants; the median ISI is 10 s. (**A**) The distribution of all ISIs. (**B**) The ISI distribution of the short ISIs from 1 to 5 s. (**C**) The ISI distribution of TMS mapping session 1.1

The range of the ISIs across TMS mapping sessions was from 1.5 to 41 s (Fig. 5A). ISIs shorter than 5 s (Fig. 5B) comprised 14% of the whole distribution, longer than 25 s – 3% of the entire distribution (Fig. 5A). The distribution of ISIs was non-normal, with a long tail towards longer ISIs for all five muscles (Fig. 5, Shapiro–Wilk test: *W* = 0.88–0.98, *p* < 0.001). The first TMS session (session 1.1, Fig. 5C) had the shortest ISIs (1.7–37.9 s, median – 5 s), while for all other three TMS mapping sessions the stimulation of the initial points was repeated based on the first session, and, thus, the ISIs were longer (session 1.2–1.9–40.2 s, median = 10.1 s; session 2.1–2.8–41.1 s, median = 10.6 s; session 2.2–3.2–41 s, median = 11.9 s) because for each stimulation point an operator had to spend time on retargeting for the precise coil location and orientation. After the log-transformation, the distribution of ISI for each dataset for APB, FDI, ADM, EDC, and BB was still non-normal (all five muscles: *W* = 0.95–0.98, *p* < 0.001).

#### MEP Amplitudes Inside the MCR

MEP amplitudes for all five muscles were non-normally distributed (APB median = 171 μV; FDI median = 267 μV; ADM median = 143 μV; EDC median = 167​​​​​​​ μV; BB median = 127​​​​​​​ μV), and even after log-transformation, the distribution remained non-normal (all five muscles: *W* = 0.98–0.99, *p* < 0.001).

#### MCR Areas

MCR areas also had non-normal distributions: APB (*W* = 0.95, *p* = 0.001), FDI (*W* = 0.96, *p* = 0.01), ADM (*W* = 0.96, *p* = 0.004), EDC (*W* = 0.96, *p* = 0.002), and BB MCR area (*W* = 0.92, *p* < 0.001). The median values for the MCR area were the following: APB – 5.96 cm^2^, FDI – 6.97 cm^2^, ADM – 5.52 cm^2^, EDC – 5.7 cm^2^, BB – 3.39 cm^2^. As the stimulation intensity was determined as 110% of APB resting motor threshold and was not adjusted for proximal muscles, as a result, in 56% of sessions, the BB MCR area was less than ¼ of the maximal BB MCR area value (17.2 cm^2^). In 4 out of 26 subjects, the BB MCR area was 0 cm^2^; therefore, we decided to exclude BB data from further analysis.

### Association Between ISI and TMS Output

#### ISI and MEP Amplitudes

We found a small but significant positive relationship between the ISIs and the MEP amplitudes for APB, ADM, FDI, and EDC across all TMS mapping sessions (see Table 1; the results for each muscle are visualised in the Supplementary material, Fig. 1S). The most pronounced slope (*β* = 0.13) and the highest explained variance (*R*^2^ = 0.12) were observed for ADM. The effect size was small for APB, FDI (both ${f}^{2}$ = 0.05), and EDC (${f}^{2}$ = 0.08), and for ADM it was closer to medium (${f}^{2}$ = 0.11).

**Table 1:**
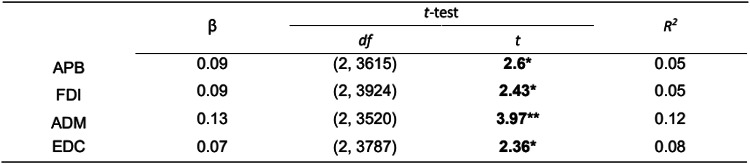
Linear Mixed-Effect Models for log-transformed MEP amplitudes and ISI

Both MEP amplitudes and ISI are log-transformed. *β* – regression coefficient in the linear-mixed effect model where ISI = β*MEP + *β*_0_ + *e*, *df–* degrees of freedom, *t* – *t*-test value, *R*^2^ – coefficient of determination,*p < 0.05; **p < 0.001.

Short ISIs comprised 14% of all the trials for each muscle. After excluding these short ISIs (less than 5 s), we observed a similar relationship between ISI and MEP for the APB and ADM muscles (see Table 2), but not anymore for FDI and EDC muscles. In addition, the changes of models’ estimates when the ISI range is shortened using 5 5-s steps are available in the supplementary material (see Fig. 3S).

**Table 2:**
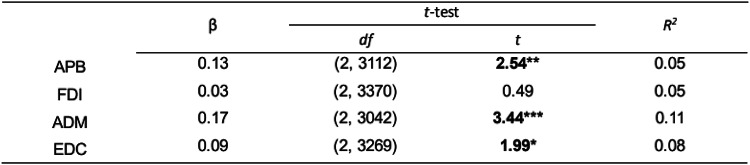
Linear Mixed-Effect Models for log-transformed MEP amplitudes and ISI after removal of the short ISI

Both MEP amplitudes and ISI are log-transformed. *β* – regression coefficient in the linear-mixed effect model where ISI = β*MEP + *β*_0_ + *e*, *df–* degrees of freedom, *t* – *t*-test value, *R*^2^ – coefficient of determination. *p < 0.05; **p < 0.01; ***p < 0.001.

When we used the 1 st PCA component from all the non-zero responses (which explained 62.56% of the total MEP variance) to exclude the muscle-specific effect, Spearman’s rank correlation coefficient revealed a weak positive correlation between this 1 st component and trial-by-trial ISI (*r* = 0.10, *p* < 0.001).

The permutation test for linear mixed-effect models for MEP and ISI revealed a significant positive relationship only for ADM and EDC in session 2.2 (see Table 3). However, when pooling data across all sessions, a consistent positive relationship between ISI and MEP amplitude emerged across all muscles.

**Table 3:**
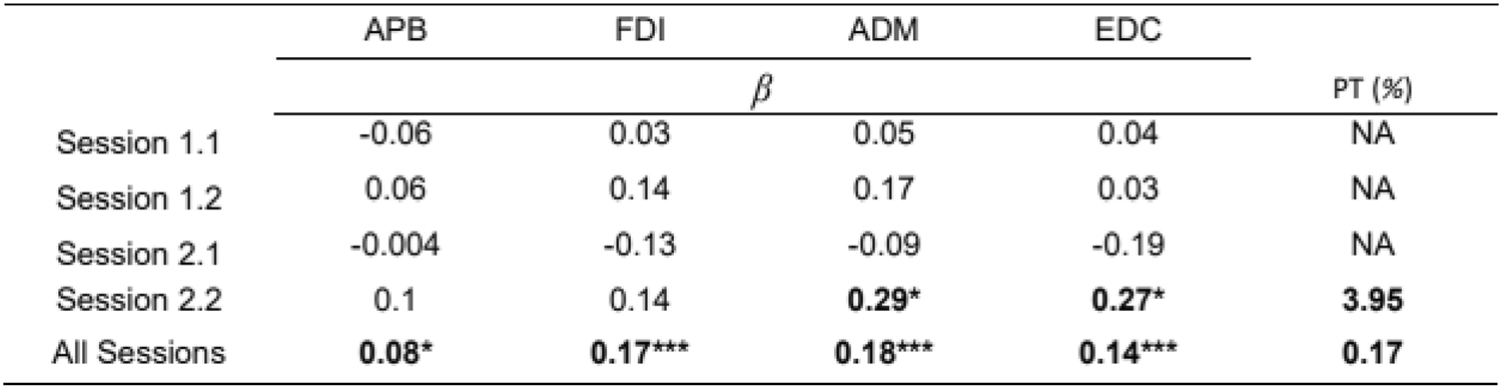
Permutation Test for Linear Mixed-Effect Models between ISI and MEP Amplitudes for each Stimulation Point at which activation of All Muscles was obtained

*β* – regression coefficient in the linear-mixed effect model where ISI = β*MEP + *β*_*0*_ + *e*. Permutation test (PT) is the proportion of sampled permutations where significant relationships between variables were equal to or stronger than the correlation observed in the original sample. PT values less than 5% mean the rejection of the null hypothesis of the random nature of the variation in the original sample. Bolded values reflect the results'significance, *p < 0.05; **p < 0.01; ***p < 0.001.

#### ISI and TMS Mapping Procedure

##### ISI and Distance Between the Successive Stimulation Points in the MCR

There was a weak but significant association between ISI and the distance between the two successive stimulation points in the TMS mapping session (please see Supplementary material, F1S). The linear mixed-effect model revealed that bigger distances between the successive stimuli were associated with longer ISI (subject as a random factor: ISI = 0.45 * Distance + 9.07, *R*^2^ adjusted = 0.10, with main effect *β* coefficient: *t* (2, 7562) = 5.18, *p* < 0.001).

##### ISI and Number of Stimulation Points Per Session

There was a significant positive correlation between the number of stimuli and the median ISI for session 1.2 (*r* = 0.6, *S* = 1184.4, *p* < 0.01) and session 2.2 (*r* = 0.62, *S* = 1100.4, *p* < 0.001). There was also a trend-level relationship for TMS session 1.1 (*r* = 0.35, *S* = 1896.6, *p* = 0.078).

##### ISI and Stimulus Counting Number (Time During the Experiment)

There was also a very weak positive association between the stimulus counting number in the session and the ISI in three out of four TMS simulation sessions (see Table 4), indicating that the ISI slightly increased towards the end of the recording.

**Table 4:**
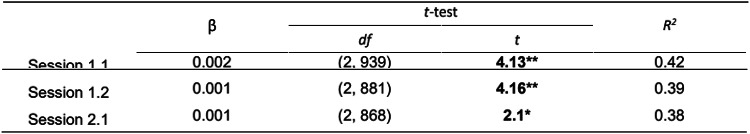
Linear Mixed-Effect Model for Stimulus Counting Number and ISI

* β* – regression coefficient, *df–* degrees of freedom, *t* – *t*-test value, *R*^2^ – coefficient of determination, *p < 0.05; **p < 0.001.

### Relationship between ISI and MCR area

#### Correlation Between MCR Area and ISI

For three TMS mapping sessions, Spearman Rank Correlation analysis and the permutation test showed significant moderate positive correlations between ISI medians and MCR’s areas (see Table 5).

**Table 5:**
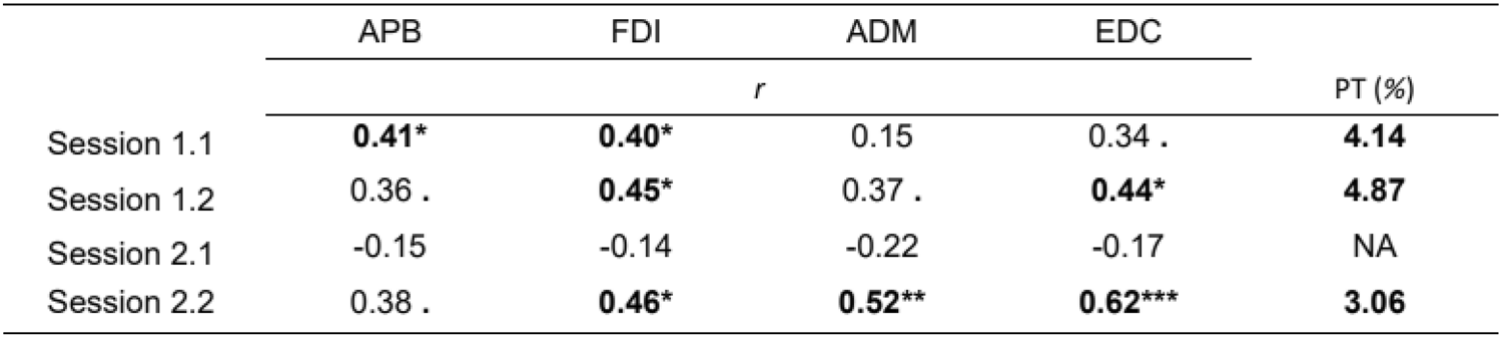
Permutation Test for Spearman Rank Correlation Analysis between ISI Medians and MCR Areas

*r* is the nonparametric measure of rank correlation, and the permutation test (PT) is the proportion of sampled permutations where significant relationships between variables were similar to the results of correlation tests in the original sample. PT values less than 5% mean the rejection of the null hypothesis of the random nature of the variation in the original sample. Bolded values reflect the results'significance, *p* < 0.1; *p < 0.05; **p < 0.01; ***p < 0.001.

#### Partial Correlation Between MCR Area and ISI While Controlling for the TMS Mapping Procedure Parameters

When controlling for the number of stimuli, there still was a significant positive correlation between MCR area and median ISI for session 2.2 for one muscle, EDC MCR area (*r* = 0.45, *p* = 0.03) (Spearman’s rank correlation coefficient). When controlling for the median distance between the two successive stimulation points in the TMS mapping session, there was also a significant positive correlation for some MCR areas and median ISIs in 3 out of 4 sessions (see Table 6). When controlling for both (1) the number of stimuli per session and (2) the median distance between the two successive stimulation points in the TMS mapping session, partial Spearman’s rank correlation for the MCR area and median ISI continued to be significant for TMS mapping session 2.2 for EDC muscle (*r* = 0.45, *p* = 0.03).

**Table 6:**
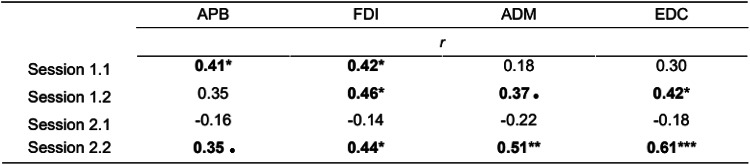
Partial Spearman’s rank correlation for MCR area and ISI when controlling for the median distance between the successive stimulation points

*r* – a nonparametric measure of rank correlation, *p* < 0.1; *p < 0.05; **p < 0.01; ***p < 0.001.

**Fig. 6:**
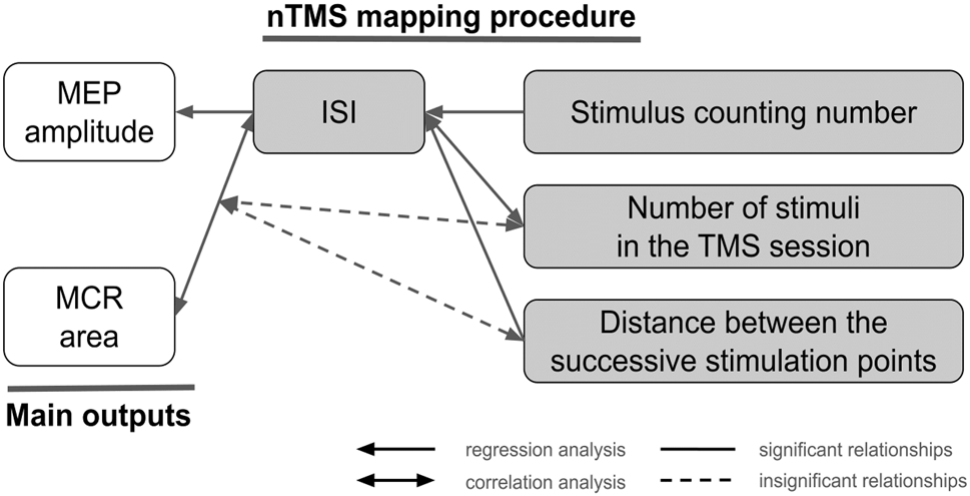
A scheme showing the results of the analyzed relationships between the main TMS outputs and the nTMS mapping procedure parameters. ISI – interstimulus interval, MEP – motor evoked potential, and MCR – muscle cortical representation. One-sided arrows indicate the direction from the independent to dependent variables in the regression analysis, and double-sided arrows indicate the correlational relationship between the variables. Solid lines denote significant relationships, and dashed lines – insignificant relationships

The summary of the results is represented in Fig. 6.

## Discussion

Our main findings are the following. We observed a weak positive association between the ISI and MEP amplitudes in all four analyzed upper limb muscles` MCRs, even though the major part of the ISIs was longer than 5 s. There was also a positive link between the median ISIs and MCR areas. Additionally, we observed a weak positive association between the ISI and TMS mapping procedure parameters: (1) distances between the successive stimulation points, (2) number of stimuli in the session, and (3) stimulus counting number in the TMS mapping session, which we accounted for.

### Association Between ISI and MEP Amplitudes

We observed a weak but significant association between ISI and trial-by-trial MEP amplitudes. The effect size for this relationship was mostly small. Multiple earlier studies using TMS applied to a single cortical spot have reported an increase in MEP amplitude when the ISI increases (Julkunen et al. 2012; Vaseghi et al. 2015; Hassanzahraee et al. 2019; Matilainen et al. 2022). Our findings are particularly intriguing because, despite the fact that MEP amplitude at each stimulated point primarily depends on the spatial location of the stimulation, we still detected the ISI effect. To our knowledge, there are no reports about the trial-by-trial MEP amplitude changes associated with ISI during TMS mapping. Even for single-pulse TMS applied to a single cortical spot, the association between the MEP amplitude and ISI requires further understanding. It could be suggested that the effects of single-pulse TMS applied using ISIs shorter than 5 s might share similar mechanisms with the low-frequency rTMS (Julkunen et al. 2012; Matilainen et al. 2022), which is believed to induce long-term depression-like effects (Ferro et al. 2022). While in some studies, the number of stimuli per session might have simply been insufficient to cause low-frequency rTMS (e.g., 31 stimuli in (Julkunen et al. 2012)). In our current study, we performed up to 284 stimuli per nTMS mapping investigation, which is similar to the amount used in some low-frequency rTMS applications (Kito et al. 2008). However, if shorter ISI induced the same process as the low-frequency rTMS, MEP amplitudes should have reached a plateau for the ISI longer than 5 s, while we observed an increase in MEP amplitude for ISI up to 41 s. In addition, even after removing the short ISIs, which constituted 14% of the trials, the association remained significant for APB and ADM.

Another possible explanation of the MEP amplitude increase, together with the ISI increase, involves the non-neuronal effects of TMS. The information on such effects is currently very limited (Cullen and Young 2016; Petrovskaya et al. 2023). Animal models of neuro-psychiatric conditions suggest possible effects of the low-frequency rTMS primarily on astrocytes, suggesting an increase of glial fibrillary acidic protein (GFAP) and astrocyte migration (Ferreira et al. 2024). However, among the studies using healthy animal models, only one paper observed an effect of the low-frequency rTMS on GFAP (Ferreira et al. 2024). In non-lesioned animals, low-frequency rTMS has been suggested to increase intracellular calcium levels, associated with increased astrocyte activity (Ferreira et al. 2024; Clarke et al. 2017, 2021). Another non-neuronal explanation of the MEP amplitude dependence on the ISI involves cerebral hemodynamics. A drop of oxyhemoglobin several seconds after a single-pulse TMS, which can be associated with neuronal excitability changes, was reported in several works (Mochizuki et al. 2006; Thomson et al. 2011; Curtin et al. 2019 for review). We believe that the hemodynamic effect may be one of the plausible explanations in our case, considering that a major part of our ISI distribution was beyond 5 s.

One possible explanation for the ISI and MEP relationship is that with longer ISIs, participants were in a state of stimulus expectation. Recently, it has been shown that the anticipatory effects can influence MEP amplitudes (Tran et al., 2021). However, authors showed a decrease in MEP amplitudes when participants were expecting a TMS pulse. Although our case is not directly comparable since our participants did not know when the TMS pulse would be delivered and could only anticipate it with the longer ISI, we observed an increase in MEP amplitudes. Therefore, the stimulus expectation does not seem to be a convincing interpretation for our results.

We observed slightly different effect sizes for the ISI-MEP link for different muscles. While APB, FDI, and EDC had a small effect, the effect for ADM was close to medium. While there is a correlation among different muscles'responses to TMS (Schabrun & Ridding 2007; Mirdamadi et al. 2016; Neva et al. 2017), their excitability patterns are different (Thordstein et al. 2013; Naish & Obhi 2015; Menon et al. 2018). In agreement with the previous literature, our findings suggest that the ISI effect on MEP may vary among muscles.

### Relationship Between ISI and MCR Area

We found a moderate positive association between the ISIs and MCR areas despite using rather long ISIs and paying particular attention to not stimulating the same cortical spot twice in a row. Even when controlling for the TMS mapping procedure parameters, such as the number of stimuli and median distance between the two successive stimulation points, the correlation between ISI and MCRs was observed in the majority of sessions. As we mentioned in the introduction, there is currently a lack of agreement regarding the use of ISIs during TMS motor mapping. Some groups performed fast TMS mapping with ISIs shorter than 5 s and suggested an ISI of 1–2 s as optimal (Van De Ruit et al. 2015; Cavaleri et al. 2018; Grab et al. 2018). At the same time, many recent TMS mapping studies with a human operator still deliberately avoided short ISIs (Malcolm et al. 2006; Reijonen et al. 2020; Nazarova et al. 2021; Weise et al. 2023). Notably, in numerous TMS mapping papers, ISIs are not commonly reported (Potter-Baker et al. 2016; Raffin and Siebner 2019; Eibl et al. 2022), whereas in the TMS-EEG field, ISIs are commonly reported and tend to vary from 1 to 6 s (Zazio et al. 2021; Zazio et al. 2022a, b; Guidali et al. 2023; Beck et al. 2024). Novel technologies, such as robotized TMS (Kantelhardt et al. 2010; Grab et al. 2018; Giuffre et al. 2021; Kahl et al. 2022) and multi-locus TMS (Koponen et al. 2018; Nieminen et al. 2022; Navarro de Lara et al. 2023), are making the operator's role in TMS mapping less critical and thus, theoretically, allowing performing TMS mapping faster (Harquel et al. 2016; Souza et al. 2022). However, longer ISI use will still be inevitable in some situations, especially in clinical or pediatric populations, when subjects have difficulty relaxing or staying still during the TMS procedure. Thus, investigating the characteristics of a traditional TMS mapping protocol with longer ISIs remains relevant.

### Limitations

Our study has limitations. First, the effect size for the association between ISI and MEP was clearly small. Second, ISIs were associated with the parameters of the TMS mapping procedure: the distance between the successive stimulation points and the overall number of stimuli in the session. However, controlling for these parameters did not entirely eliminate the ISI effect on MCR areas. Fourth, the first TMS mapping session differed from the other TMS sessions. During these last three sessions, the targets stimulated in the initial session were revisited using the navigation system, resulting in longer ISIs. Lastly, we explored only a highly homogeneous population of young right-handed males. Future research involving more heterogeneous groups, including females and older participants, is needed to further elucidate the effects of ISI on TMS motor mapping.

### Outlook

Currently, there is no consensus about the optimal ISI during TMS mapping. Our study has revealed a weak effect of ISIs on MEP amplitudes and MCR areas in a comprehensive nTMS motor mapping using ISIs for up to 41 s. However, while the small effect of ISI may be safely disregarded if the studied effects on MEP are pronounced, in cases when small effects are tested, ISI should be considered more carefully so as not to mask the target effect. This can be particularly important for TMS mapping studies probing the changes during training or rehabilitation. In addition, since we observed slight variations among muscles in the association between ISI and MEP, it is important to consider differences in muscle excitability. We recommend reporting ISI not only in studies focused on TMS motor mapping but in general, in all TMS studies investigating cortical or corticospinal excitability patterns.

## Supplementary Information

Below is the link to the electronic supplementary material.

Supplementary file1 (DOCX 1119 KB)

## Data Availability

The collected human data cannot be efficiently anonymized and thus will not be openly released; yet, the custom-made software for TMS mapping analysis is free and publicly available at http://tmsmap.net/. Data supporting the findings of this study will be also available from the corresponding author upon reasonable request.
