# Supplementary material for: On the Relation Between the Interstimulus Intervals and Multi-Muscle nTMS Motor Mapping
Source: Brain Topogr. 2025 Jul 30;38(5):55. doi: 10.1007/s10548-025-01128-9 (PMC12310799; doi:10.1007/s10548-025-01128-9)
Supplement: Supplementary file 1 — Supplementary file1 (DOCX 1119 KB) [file 10548_2025_1128_MOESM1_ESM.docx]

**On the relation between the interstimulus intervals and multi-muscle nTMS motor mapping**

A. Asmolova*, A. Sukmanova*, M. Makarova, P. Novikov, V. Nikulin and M. Nazarova

Anastasiia Asmolova
Max Planck School of Cognition, Leipzig, Germany

Max Planck Institute for Human Cognitive and Brain Sciences, Leipzig, Germany

asmolova@cbs.mpg.de

0000-0003-1789-754X

Anastasiia Sukmanova

HSE University, Centre for Cognition and Decision making, Moscow, Russia

anastasiya.sukmanova@gmail.com

0000-0001-5218-7012

Milana Makarova

m.makarova@hse.ru

HSE University, Center for Bioelectric Interfaces, Moscow, Russia

0000-0002-9351-6588

Pavel Novikov

HSE University, Centre for Cognition and Decision making, Moscow, Russia

novikov.engineer@gmail.com

0000-0003-4102-1580

Vadim Nikulin

Max Planck Institute for Human Cognitive and Brain Sciences, Leipzig, Germany

nikulin@cbs.mpg.de

0000-0001-6082-3859

Maria Nazarova

Max Planck Institute for Human Cognitive and Brain Sciences, Leipzig, Germany

chantante@gmail.com

0000-0001-5347-5948

Supplementary material


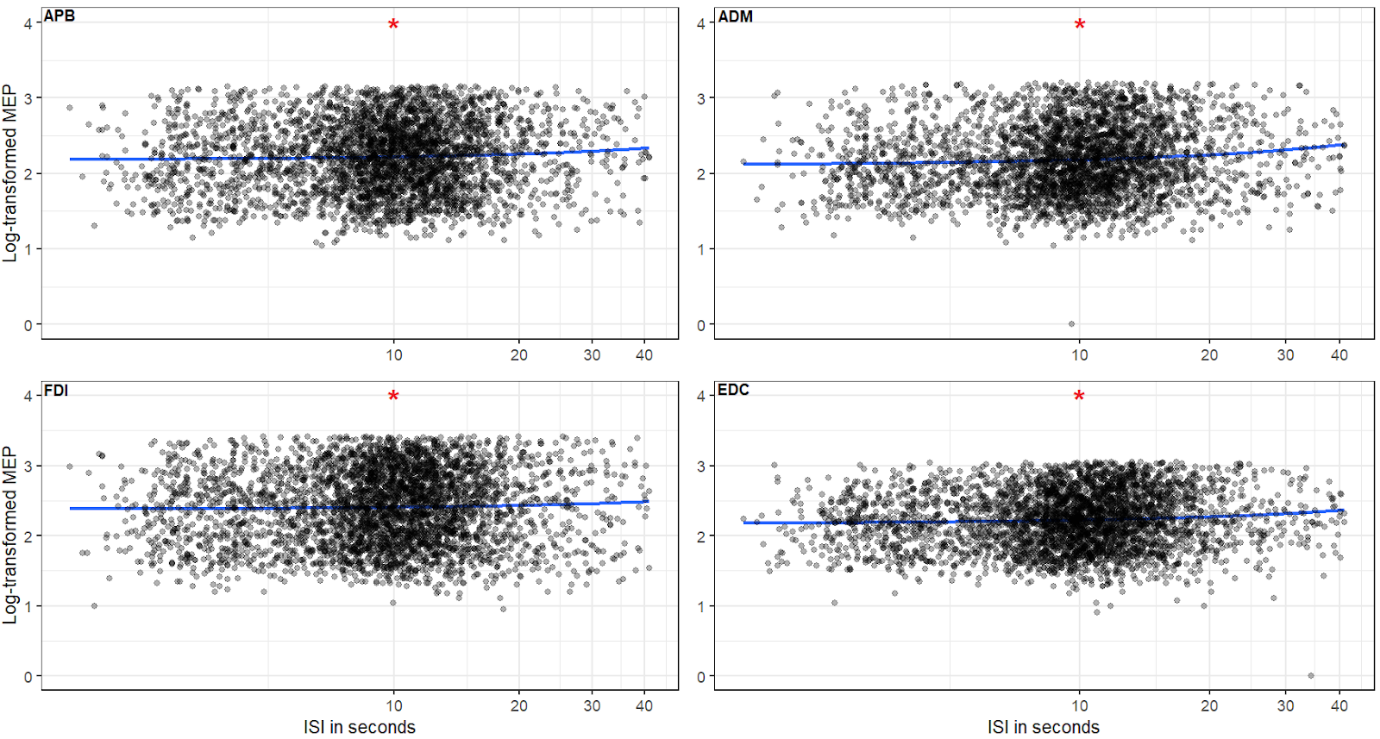


Figure 1S. Linear relationship between the trial-by-trial ISIs and log-transformed MEP peak-peak amplitudes of APB, ADM, FDI, and EDC plotted on a logarithmic x-axis. All relationships are significant. Each dot represents one trial.


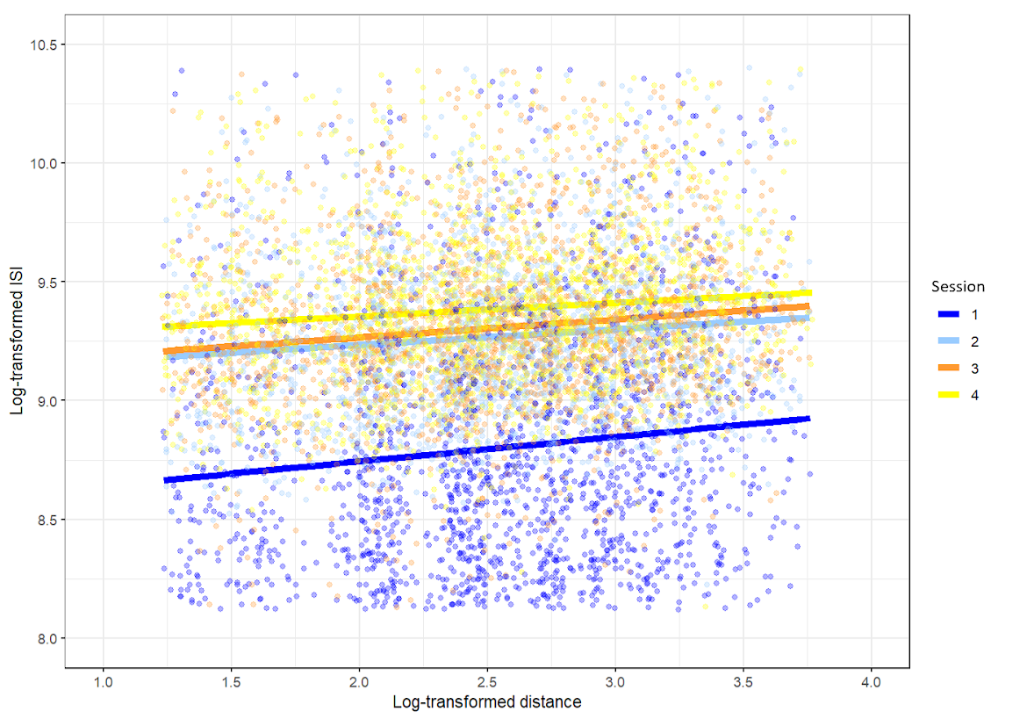


2.1

1.2

2.2

1.1

Figure 2S. The relationship between the distances between the successive stimulation points and ISIs. Colors represent different TMS mapping sessions.


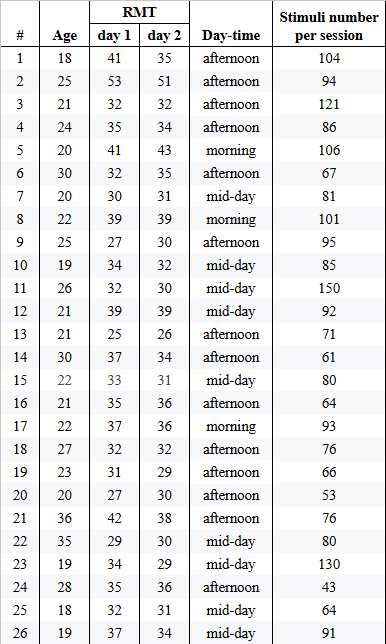


Table 1S. The individual RMT values in % of the maximum stimulator output.


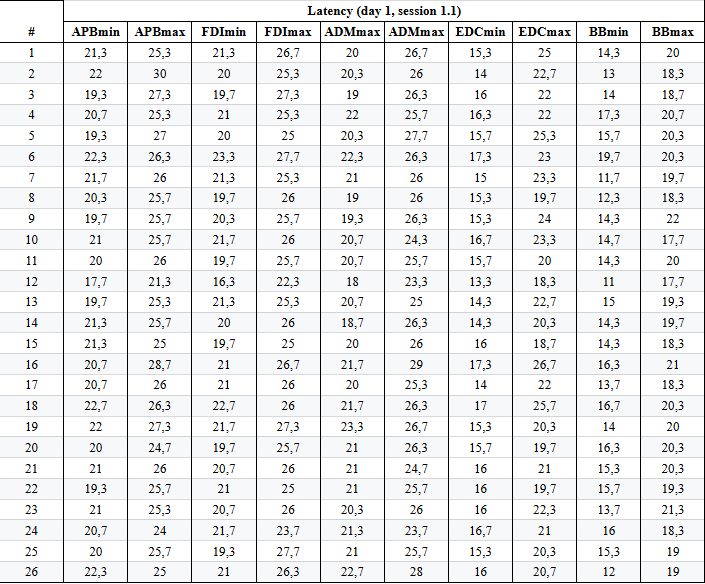


Table 2S. The minimal and maximal latencies of MEPs in milliseconds per muscle for session 1.1.


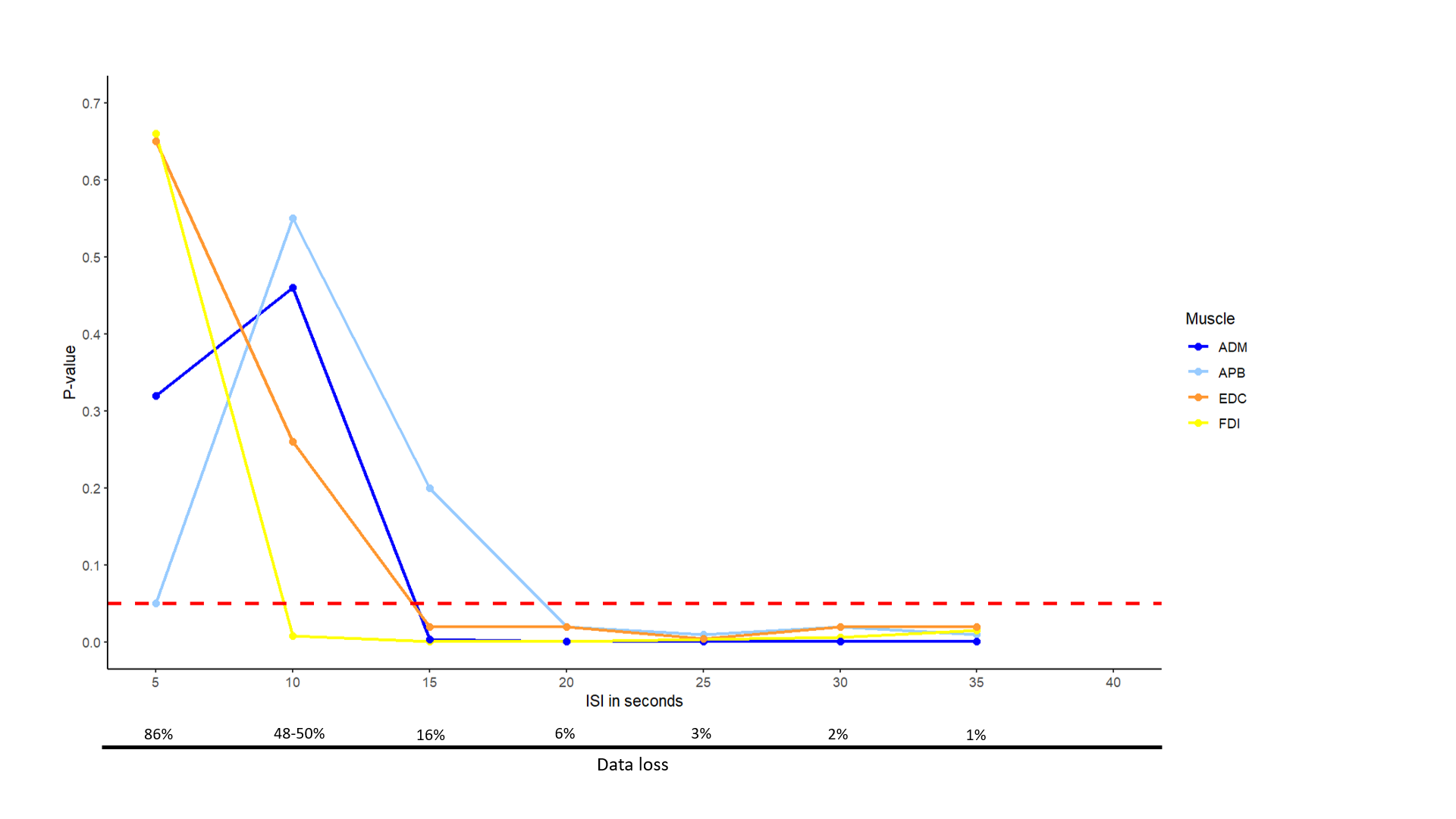


Figure 3S. Change of p-values while limiting the ISI range. On the x-axis, ISI values in seconds that were used as a limit for MEP~ISI models, on the y-axis, p-values for the regression coefficient in each model. Each muscle's data are color-coded. The dashed line is for a p-value of 0.05. The lower panel represents the percentage of data loss from the original datasets for each muscle (e.g., when ISI is limited to 35 seconds, only 1% of trials from the original dataset are removed). Since the data for each muscle was analysed separately, the % of data loss is slightly different at 10 seconds (APB and FDI - 48%, ADM, EDC - 50 %).
